# Successful management and 10-year survival of a rectal neuroendocrine tumor with rare systemic metastases via a non-portal venous pathway

**DOI:** 10.1007/s12328-026-02295-3

**Published:** 2026-03-17

**Authors:** Shigeru Horiguchi, Kazuyuki Matsumoto, Kazuya Miyamoto, Akihiro Matsumi, Hiroyuki Terasawa, Yuki Fujii, Kosei Takagi, Takashi Kuise, Takehiro Tanaka, Motoyuki Otsuka

**Affiliations:** 1 Department of Gastroenterology and Hepatology, Okayama University Hospital, 2-5-1 Shikata-cho, Kita-ku, Okayama-city, Okayama 700-8558 Japan; 2 Department of Gastroenterological Surgery, Dentistry, and Pharmaceutical Sciences, Okayama University Graduate School of Medicine, Okayama, Japan; 3 Department of Gastroenterological Surgery, Okayama Red Cross Hospital, Okayama, Japan; 4 Department of Pathology, Okayama University Hospital, Okayama, Japan

**Keywords:** Rectal neuroendocrine tumor, Rare metastases, Multimodal treatment

## Abstract

Metastatic neuroendocrine tumors (NETs) typically involve the liver and lymph nodes. Metastases in the orbit, heart, and ovary are rare and present unique clinical challenges. We report a woman who was 70 years old at the time of initial presentation with liver metastases from a rectal neuroendocrine tumor. Following a right hepatectomy for liver metastases, she remained free of hepatic recurrence for 10 years. Notably, four years after the right hepatectomy, she developed new metastases in the left orbit, right ventricle, and left ovary, with diplopia as the sole clinical symptom. This clinical course suggests a distinct metastatic pathway associated with the lower rectum that bypasses the portal circulation, consistent with the dual drainage system of the rectal region. Management involved a strategic multimodal approach that includes systemic therapy with everolimus and lanreotide as well as targeted surgical resectioning of the cardiac and ovarian lesions. The orbital lesion achieved a complete response through systemic therapy alone, preserving visual function. Currently, 10 years after the initial treatment, the patient maintains an excellent performance status (ECOG PS 0) while receiving peptide receptor radionuclide therapy (PRRT). This case demonstrates that recognizing atypical metastatic pathways and employing strategic multimodal therapies can achieve long-term survival and functional preservation in rectal NETs.

## Introduction

Neuroendocrine tumors (NETs) primarily metastasize to the liver and lymph nodes through hematogenous and lymphatic pathways. However, metastases to unusual sites such as the orbit, heart, and ovaries are rare clinical entities that pose significant therapeutic challenges. Orbital involvement is infrequent, with a reported incidence of approximately 2.7% [1], and cardiac metastases occur in only 1.5–2.4% of cases [2, 3]. These atypical presentations require a delicate balance between oncological control and the preservation of organ function and quality of life.

Current clinical evidence, including the RADIANT-4 trial [4] and EVERLAR study [5], supports the efficacy of everolimus and somatostatin analogs for systemic control of advanced NETs. In this study, we report a case of a rectal NET with these rare systemic metastases and discuss the strategic treatment process and the presumed underlying metastatic mechanisms.

## Case report

A 70-year-old woman was referred to our hospital after multiple liver tumors were detected during a routine health examination. She had no specific symptoms at presentation. Based on liver biopsy findings, she was diagnosed with NET G2 liver metastases. Her medical history included an untreated left pharyngeal pleomorphic adenoma, which was confirmed on review at our pathology department. Additionally, a cardiac pacemaker was inserted to treat a complete atrioventricular block.

**Fig. 1:**
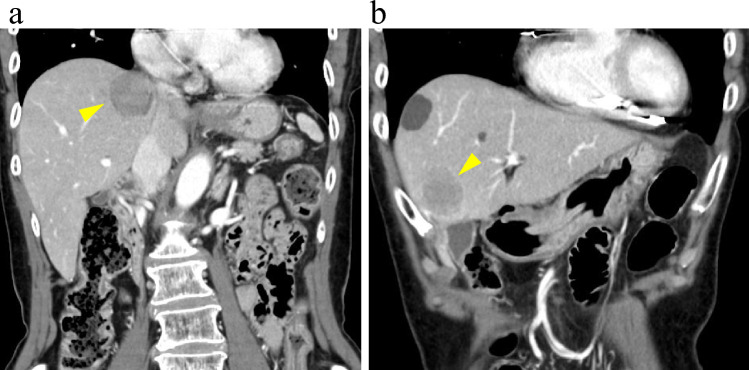
Dynamic computed tomography (CT) images at the arterial phase upon referral to our hospital. **a** A coronal image showing a low-attenuation area in segment 7 of the right hepatic lobe (arrowhead). **b** An axial image showing a low-attenuation area in segment 5 of the right hepatic lobe (arrowhead)

Initial evaluation with upper and lower gastrointestinal endoscopy and contrast-enhanced CT failed to identify the primary lesion. As the liver metastases were confined to the right lobe (Fig. 1a, b, arrowheads: tumor area), right hepatectomy was performed with curative intent. Histopathological examination revealed tumor cells with round to oval nuclei arranged in trabecular and insular patterns, indicating expansive growth. Immunohistochemically, the tumor was positive for synaptophysin and negative for chromogranin A. With a Ki67 labeling index of 20.6%, the diagnosis was revised to NET G3.

Post-hepatectomy follow-up imaging showed no evidence of new lesions. However, 68 Ga-DOTATOC PET/CT, performed to evaluate potential residual disease at another hospital, revealed an uptake in the perirectal region and at the site of the known left pharyngeal pleomorphic adenoma. Subsequently, a detailed medical history of the perirectal lesion revealed that the patient had undergone endoscopic mucosal resection (EMR) of a rectal carcinoid tumor at a local clinic 10 years prior to the current presentation. Review of the endoscopic images from the initial EMR revealed a tumor measuring 17 × 15 mm, with pathological findings indicating uncertainty regarding the completeness of resection. Although the original specimen was unavailable for WHO 2019 classification review, we clinically diagnosed the liver lesions as metastases from the rectal NET. Contrast-enhanced CT revealed a 5-mm lymph node corresponding to the perirectal uptake area identified on DOTATOC-PET/CT, which was considered to be local recurrence after EMR.

Given the recurrence of NET in both local and liver sites 10 years after initial endoscopic treatment, we initiated systemic therapy before rectal and perirectal lymph node resection. Specifically, daily treatment with 10 mg of everolimus was initiated. After 12 months of stable disease, the patient underwent laparoscopic intersphincteric resection with D2 lymph node dissection and lateral pelvic node dissection. Pathological examination revealed NET metastases in lymph nodes #251 (Ki67 20.5%, NET G3) and #263 (Ki67 15%, NET G2); however, no residual tumor was found in the rectum.

**Fig. 2:**
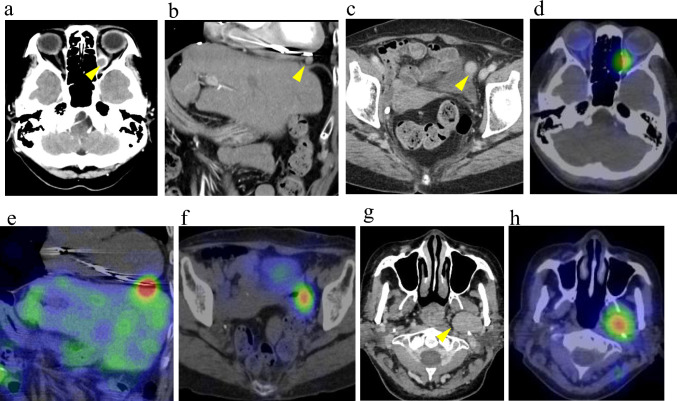
Imaging findings of the systemic lesions. **a**–**c** Dynamic CT images: **a** Head axial view, **b** chest and abdomen coronal view, and **c** pelvis axial view. Arrowheads indicate an enhancing mass in the left medial rectus muscle, a small nodule adjacent to the right ventricle, and a faintly enhancing nodule in the left ovary, respectively. **d**–**f** SRS: Images correspond to sections (**a**), (**b**), and (**c**). Each lesion identified on CT shows somatostatin receptor expression. **g** Enhanced head CT (axial view): The arrowhead indicates a known pleomorphic adenoma. **h** SRS (head, axial view): Significant uptake is noted in the pleomorphic adenoma, corresponding to the CT finding in **g**

**Fig. 3:**
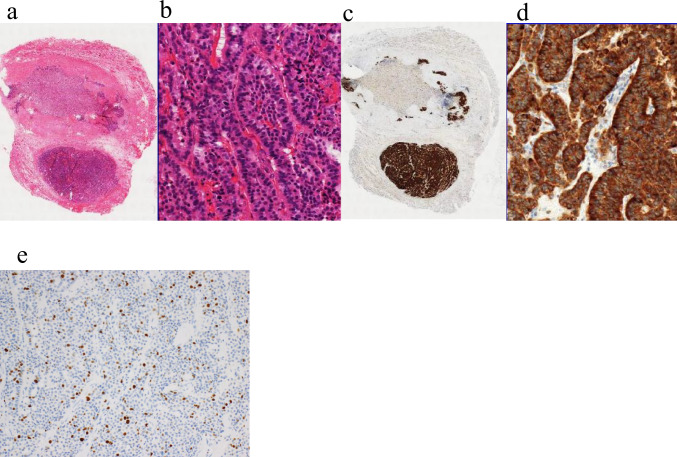
Histopathological findings of the orbital mass biopsy. **a** Low-magnification hematoxylin and eosin (H.E staining) image: An overview of the biopsy specimen. **b** High-magnification H.E image: The tumor shows nested growth of relatively uniform cuboidal epithelial cells. **c** Synaptophysin staining: Low-magnification image of the section corresponding to (**a**). **d** High-magnification image of Synaptophysin staining **e** Ki-67 immunohistochemistry: The Ki-67 labeling index is 19.8%

Two years and five months after rectal surgery, the patient developed diplopia. Contrast-enhanced CT revealed a tumor in the left-orbital medial rectus muscle region, a lesion adjacent to the right ventricle, and an ovarian lesion. (Fig. 2a, b, c, arrowheads: tumor area). Somatostatin receptor scintigraphy (SRS) showed an uptake by these lesions (Fig. 2d, e, f) and by the known pharyngeal pleomorphic adenoma (Fig. 2 g, arrowheads: tumor area, 2 h). To determine whether the orbital lesion originated from a NET metastasis or from the known pharyngeal pleomorphic adenoma [6], orbital biopsy was performed, confirming NET metastasis (Ki67 19.8%, NET G2; Fig. 3a, b, c, d, e).

**Fig. 4:**
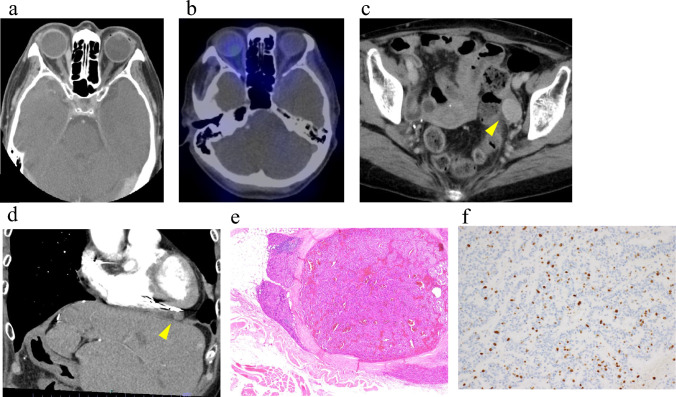
Follow-up imaging and histopathological findings after initiation of everolimus and lanreotide. **a** Head CT (axial view): The previously observed tumor in the left medial rectus muscle has disappeared. **b** SRS (head, axial view): Corresponding to (**a**), no somatostatin receptor uptake is identified in the orbital region. **c** Enhanced CT of the pelvis: The ovarian lesion shows a clear increase in size (arrowhead). **d** Enhanced CT of the chest and abdomen: The lesion adjacent to the right ventricle shows a slight tendency toward enlargement (arrowhead). **e** Histopathology of the cardiac lesion (H.E staining): The tumor has invaded the right ventricular myocardium. It shows nested growth of cuboidal tumor cells with subround nuclei, consistent with metastasis from a neuroendocrine tumor. **f** Ki-67 immunohistochemistry: The Ki-67 labeling index is 15%

Considering the multiple metastatic sites and the need to preserve visual function, systemic therapy with everolimus and lanreotide was initiated. The decision to combine these agents was based on the development of new metastases despite prior everolimus treatment and the positive octreotide uptake in the lesions. Tumor response was evaluated according to RECIST version 1.1 criteria after three months of treatment. The orbital lesion showed a complete response (Fig. 4a, b), the right ventricular mass remained stable, and the ovarian lesions progressed (Fig. 4c). Surgical resection of the ovarian metastases revealed NET G3 (Ki67 30.1%). Four months after ovarian surgery, the right ventricular mass showed slight progression (Fig. 4d). Cardiac surgery achieved complete resection of the lesion, which was confirmed as NET G2 (Ki67 15%) (Fig. 4e, f).

Six months after cardiac surgery, the patient developed pancreatic, adrenal, and bone metastases. Treatment was switched to PRRT, which became available in Japan at that time. After three years of PRRT, the patient continues to display a complete response of the orbital lesion, no recurrence at ovarian or cardiac sites, and stable disease in other metastatic sites, while maintaining ECOG PS 0 and continuing regular work activities. Throughout the 10-year clinical course following the initial right hepatectomy, the patient remained free of recurrent liver metastases.

## Discussion

Orbital metastasis of NET is rare, with a reported incidence of approximately 2.7% in a large-scale database analysis [1]. The primary sites are most commonly located in the gastrointestinal tract, particularly the midgut (ileum), and the lungs (Table 1) [1, 7–11]. Clinically, these metastases often present with symptoms such as proptosis, diplopia, and decreased vision (Table 1), owing to their tendency to occur in the extraocular muscles. In the present case, contrast-enhanced CT and SRS were instrumental in detecting the orbital lesion. Notably, our patient had remained free of liver recurrence for over 10 years following liver resection, which is a highly atypical clinical course compared to most reported cases where orbital involvement occurs alongside active liver metastases (Table 1). This unusual presentation suggests the possibility of a metastatic pathway distinct from the common portal venous route.

**Table 1:** Summary of previously reported cases of orbital metastasis from neuroendocrine tumors

Author	Age	Sex	Primary	WHO grade	Laterality	Presenting symptom	Liver metastasis*	Treatment for orbit metastasis	Survival period†
Das	72	F	Midgut (Jejunum)	G3	B	None	Absent	CAPTEM	21 years (alive)
	68	M	Midgut (Ileum)	G2	B	Periorbital swelling	Present	Radiation	6 years (dead)
	63	F	Midgut (Ileum)	G3	B	None	Present	PRRT	24 years (alive)
	72	M	Midgut (Ileum)	G2	U	None	Absent	Radiation	12 years (alive)
	61	F	Midgut (NOS)	G2	B	Diplopia	Present	Radiation + Capecitabine	8 years (alive)
Ueno	39	M	Foregut (Pancreas)	N/A	B	Ptosis	Present	Radiation	6 years (alive)
Tayon	58	F	Midgut (Ileum)	N/A	U	Diplopia, pain, conjunctival chemosis	Absent	Surgery	2 months (alive)
Mustak (N = 28)	59	M (43%) F (57%)	GI (50%), Lung (7.1%), Kidney (7.1%), Ovary (3.6%), Unknown (29%)	N/A	U (68%) B (14%)	Proptosis (71%) Diplopia (61%) Decreased vision (43%) Pain (25%) Motility restriction (68%)	N/A	Surgery (61%) Octreotide (39%) Radiation (36%) Chemotherapy (32%) Steroids (11%) Observation (14%)	5-year survival from orbital diagnosis: 50%
Kamieniarz (N = 27)	59	M (44%) F (56%)	Midgut (67%) Foregut (Pancreas) (15%) Bronchial (7.4%) MTC (3.7%) Unknown (7.4%)	G1 (42%) G2 (53%) G3 (5%)	U (93%) B (7%)	Asymptomatic (59%) Proptosis (19%) Diplopia (15%) Partial loss of vision (15%) Loss of acuity (11%) Pain (3.7%) Discomfort (3.7%) Loss of acuity on short Distance vision (3.7%)	Present (85%)	Local therapy: 11 cases (Symptomatic only) Radiation (N = 6, 55%) Biopsy and debulking (N = 3, 27%) Others (N = 2, 18%)	N/A
Hosalkar	57	M	Midgut (small intestine)	N/A	U	Diplopia, red eye, proptosis supraduction deficit	Present	Radiation	N/A
Our case	70	F	Hindgut (rectum)	G2	U	Diplopia	Absent	Lanreotide + everolimus	10 years (alive)

*M* male, *F* female

Laterality: Laterality of the orbital metastatic neuroendocrine neoplasm (*U* unilateral, *B* bilateral)

*N/A* data not available, *GI* Gastrointestinal tract, *MTC* Medullary thyroid carcinoma *PRRT* Peptide receptor radionuclide therapy. Liver metastasis*: At the time of orbital metastasis diagnosis,

Survival Period†: Time since any metastasis

Cardiac metastasis of NETs is also a rare clinical entity, with recent large-scale registry data reporting a prevalence ranging from 1.5 to 2.4% [2, 3]. The primary sites most frequently associated with cardiac metastases are the small intestine (midgut), followed by the lungs and pancreas, with myocardial lesions predominantly located in the left ventricle and the interventricular septum (Table 2) [2, 3, 12–16]. With regard to histological characteristics, there is a lack of consensus: while many reports indicate that cardiac metastases often arise from well-differentiated Grade 1 or 2 tumors [2, 16], Kunz observed that patients with cardiac metastases tended to have significantly higher Ki-67 indexes compared to those without it [3]. This suggests that while cardiac metastases can occur in indolent tumors, a higher proliferative potential may increase the risk of cardiac involvement. Notably, our case was characterized by the absence of synchronous liver metastases, a rare presentation given that cardiac involvement typically occurs in the context of advanced systemic spread. Given the Grade 2 biology and rare metastatic sites (heart and orbit), we initially attempted systemic therapy. However, the cardiac lesion’s progression despite treatment necessitated surgical resection to avert complications. The patient’s subsequent favorable outcome suggests that for localized therapy-resistant cardiac metastases, particularly in the absence of extensive liver disease, surgery is a crucial intervention for achieving long-term stability [15].

**Table 2:** Summary of previously reported cases of cardiac metastasis from neuroendocrine tumors

Author	Age	Sex	Primary	WHO grade	Location of cardiac metastasis	Symptoms of heart metastases	Liver metastasis*	Treatment for cardiac metastasis	Survival period†
Jann	59	M	Unknown	WDEC	Intramural left-ventricular	Palpitations, dyspnea, sweating, arrhythmias, chest pain	Absent	Surgery	10 years (alive)
	48	F	Midgut (Ileum)	WDEC	Right ventricular wall, interventricular septum	Asymptomatic	Present	SSA, PRRT	12 years (dead)
	44	M	Midgut (Ileum)	G1	Interventricular septum	Asymptomatic	Present	SSA	1 year (alive)
	62	M	Midgut (small intestine)	WDEC	Lateral left ventricular wall	Asymptomatic	Absent	SSA, IFN, PRRT	6 years (alive)
Kunz (N = 15)	65	M (60%) F (40%)	Midgut (small intestine) (73%) Colon (13%) Appendix (6.7%) Unknown (6.7%)	G1 (50%) G2 (30%) G3 (20%)	Left ventricle (43%) Right ventricle (14%) Septum (43%)	N/A	Present (40%)	N/A	N/A
Kinney	65	F	Midgut (Ileum)	G1	Left ventricular myocardium	Lower extremity swelling, dyspnea on exertion	Present	SSA	N/A
Mikail	36	M	Midgut (Ileum)	G1	Subepicardial layer of the anterolateral wall of the left ventricle	Asymptomatic	Present	Surgery	N/A
Clermidy	70	M	Midgut (Ileum)	G1	Intramyocardial metastasis of right ventricle free wall	Asymptomatic	Absent	Surgery	2 years (alive)
Arnfield (N = 19)	63	M (58%) F (42%)	Midgut (small intestine) (79%) Pulmonary (21%)	G1 (42%) G2 (26%) Unknown (32%)	Left ventricle (53%) Right ventricle (30%) Septum (14%) Right atrium (3%) Intramyocardial (92%) Epicardial (8%)	Asymptomatic (N = 17) Dyspnea (N = 1) Heart failure (N = 1)	Present (79%)	SSA (21%) PRRT + SSA (58%) PRRT (5%) Other (16%)	N/A
Our case	70	F	Hindgut (rectum)	G2	Epicardium of the right ventricle	Asymptomatic	Absent	Surgery	10 years (alive)

*N/A* data not available, *WDEC* well-differentiated endocrine carcinoma, *SSA* somatostatin analog, *PRRT* Peptide receptor radionuclide therapy

Liver metastasis*: At the time of cardiac metastasis diagnosis

Survival Period†: Time since any metastasis

Anatomically, the lower rectum has dual drainage pathways: the portal system and systemic circulation. In the present case, metastasis was identified in the lateral pelvic lymph node (#263) at the time of surgery for the rectal tumor. This node belongs to the lateral lymphatic drainage pathway along the internal iliac vessels, which eventually connects to the systemic circulation rather than the portal system. This involvement may indicate an early potential for systemic dissemination that bypasses the liver. Although literature on the dual drainage of rectal NETs is scarce, these anatomical pathways are well-established in rectal cancer. Given these anatomical similarities, the present clinical course, marked by the absence of liver or lung recurrence for ten years post-hepatectomy, strongly suggests that a parallel systemic route bypassing the liver contributed to the late-stage distant metastases [17–19]. As previously documented in rectal adenocarcinoma, while the portal system typically directs tumor cells to the liver [20], they can also reach the systemic circulation directly via the middle/inferior rectal veins or the valveless Batson venous plexus [19, 17]. In this case, the presence of a lateral lymph node metastasis (#263) further supports the involvement of these non-portal pathways, allowing tumor emboli to circumvent hepatic and pulmonary filtration and reach the orbit and heart.

The patient presented with diplopia caused by the orbital metastasis, necessitating rapid and robust disease control. We opted for a combination therapy of everolimus and lanreotide, based on the following three lines of clinical evidence. First, the efficacy of everolimus in non-functioning NETs was firmly established by the phase III RADIANT-4 trial [4], wherein everolimus significantly reduced the risk of progression by 52% (HR 0.48, *p* < 0.00001) and extended the median progression-free survival (PFS) to 11 months compared to placebo. Second, site-specific efficacy for the rectal primary was supported by a subgroup analysis of the RADIANT-2 study [21], which demonstrated that the combination of everolimus and a somatostatin analogue (SSA) in patients with colorectal NETs resulted in a 66% reduction in the risk of progression (HR 0.34, *p* = 0.011) as well as a remarkably prolonged median PFS of approximately 30 months. Third, the feasibility of the combination therapy in non-functioning lesions was validated by the phase II EVERLAR study [5]; this research confirmed that the everolimus and SSA combination proved safe and effective for patients with non-functioning gastrointestinal NETs, showing a favorable 12-month PFS rate of 62.3%. Based on these findings, this combination therapy was regarded as the most effective approach for managing multiple systemic metastases, which included symptomatic orbital involvement.

## Conclusion

We reported a rare case of rectal NET with orbital and myocardial metastases. This case underscores that recognizing atypical metastatic pathways and employing strategic multimodal therapies can achieve long-term survival and functional preservation. Our 10-year successful management highlights the clinical importance of considering non-portal routes in coordinating systemic and local treatments for complex rectal NET recurrences.

## Data Availability

Not applicable.
